# Epidemiology of SARS-CoV-2 Omicron BA.5 Infections, Macau, June–July 2022

**DOI:** 10.3201/eid2902.221243

**Published:** 2023-02

**Authors:** Weijia Xiong, Liping Peng, Tim K. Tsang, Benjamin J. Cowling

**Affiliations:** University of Hong Kong, Hong Kong (W. Xiong, L. Peng, T.K. Tsang, B.J. Cowling);; Laboratory of Data Discovery for Health Limited, Hong Kong (T.K. Tsang, B.J. Cowling)

**Keywords:** COVID-19, respiratory infections, severe acute respiratory syndrome coronavirus 2, SARS-CoV-2, SARS, coronavirus disease, zoonoses, viruses, coronavirus, reproductive number, public health, social measures, incubation period, Omicron variant, Macau

## Abstract

A SARS-CoV-2 Omicron BA.5 outbreak occurred in Macau from mid-June through July 2022. Out of >1,800 laboratory-confirmed cases, most were mild or asymptomatic; only 6 deaths were recorded. The outbreak was controlled through stringent public health and social measures, such as repeated universal testing and a stay-at-home order lasting 2 weeks.

The SARS-CoV-2 Omicron subvariant BA.5 has spread rapidly worldwide. A recent outbreak of BA.5 occurred in Macau during June 18–July 31, 2022. The outbreak resulted in 1,821 confirmed cases and 6 deaths but was promptly controlled. We describe the basic epidemiology of this outbreak.

Macau, a special administrative region of China with 683,000 persons, has been applying intensive public health and social measures to reduce SARS-CoV-2 variant importation and prevent community outbreaks as part of China’s dynamic zero COVID strategy. In Macau, this strategy has included stringent travel restrictions and up to 28-day on-arrival quarantines (*1*) to avoid infections within communities. As in China, all SARS-CoV-2–infected persons are strictly isolated in special facilities, and contact tracing expedites timely quarantine of close contacts outside the home. Throughout the pandemic before June 2022, only 17 domestic confirmed cases (2.5 cases/100,000 population) and no deaths were reported in Macau. Since early 2021, inactivated virus (Sinopharm, http://www.sinopharm.com) and mRNA (Pfizer-BioNTech, https://www.pfizer.com) vaccines have been available in Macau. By June 19, 2022, vaccine coverage within the entire population was 85.6% for >2 doses and 40.5% for 3 doses.

In mid-June 2022, a SARS-CoV-2 Omicron BA.5 outbreak began in Macau (*2*). The first case was detected in a person with symptoms who sought treatment at a hospital on June 18, 2022. The source of infection remains unknown (*3*). Identification of a community outbreak prompted the government of Macau to impose a series of domestic public health and social measures to control local transmission (Appendix Table 1). Macau entered an immediate prevention state at 1:00 AM on June 19, 2022. Multiple rounds of universal PCR testing were scheduled; and 14 rounds of citywide PCR testing were conducted for all persons in Macau. To identify infected persons, PCR testing was performed on June 22 and 25 for specific groups that included persons with Myanmar passports and those who sought care at places visited by persons who had SARS-CoV-2–positive tests (*4*,*5*). On June 23, schools, entertainment venues, public dining, and other nonessential businesses were closed, and residents were encouraged to stay at home. Closed-loop management was implemented in residential care homes beginning on June 25. Beginning on June 27, all persons were asked to conduct daily rapid antigen tests and report test results to an online platform (Appendix Tables 1, 2). The government enabled the risk check function of the Macau health code and implemented a district-specific epidemic prevention plan. Yellow- and red-code zones with movement restrictions were announced daily. As the daily case numbers grew, the government issued static management instructions comparable to a complete stay-at-home order beginning after midnight on July 11 and lasting until midnight on July 23; essential workers were excluded.

Among 1,821 cases that included 937 female and 884 male persons (3 months to 100 years of age), a total of 1,116 were classified as asymptomatic. The daily number of new positive cases peaked on July 5, 2022, at 146 PCR-positive cases (Figure, panel A). We estimated the time-varying reproductive number (Figure, panel B) to quantify real-time transmissibility (Appendix). The estimated time-varying reproductive number was <1 after July 7, 2022. In the final (14th) round of universal PCR testing on July 30–31, SARS-CoV-2 RNA was not found in specimens from persons in the community, confirming that the outbreak was contained.

**Figure:**
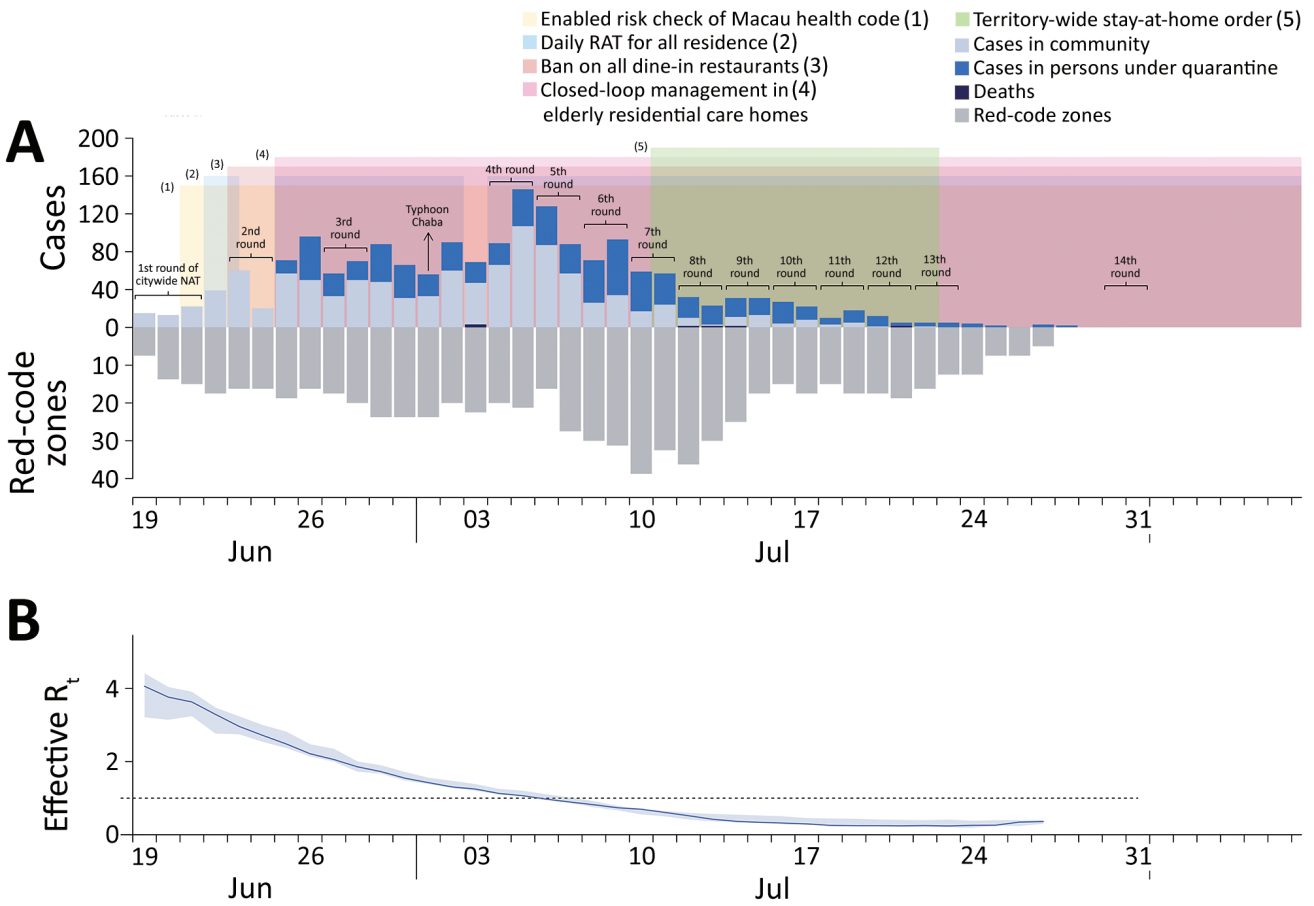
Number of PCR-positive COVID-19 cases and time-varying reproductive number in study of epidemiology of SARS-CoV-2 Omicron BA.5 infections, Macau, June–July 2022. A) PCR-confirmed COVID-19 cases and deaths in Macau during June and July 2022. Light blue bars indicate daily numbers of COVID-19 cases confirmed by PCR in the community; dark blue bars indicate persons under quarantine; black bars indicate number of reported deaths. Gray bars under the x-axis indicate the number of real-time red-code zones (areas with movement restrictions in place) in Macau. Shaded areas indicate when public health and social measures (indicated by numbers 1–5) were implemented to control COVID-19 transmission. B) Estimates of time-varying R_t_ to quantify real-time transmissibility of SARS-CoV-2 Omicron BA.5 in Macau. Dotted line indicates R_t_ of 1. RAT, rapid antigen test; R_t_, effective reproductive number

Using information on 500 case-patients with known exposure and symptom onset reported during the early outbreak phase, we estimated that the mean incubation period for Omicron BA.5 was 3.27 days (SD +1.05 days), after adjusting for exponential growth (*6*) (Table; Appendix Figure). The BA.5 incubation period was similar to 3.2 days for Omicron BA.1 (*7*) and 4.5 days for BA.2 (*8*) and shorter than that for other SARS-COV-2 variants (*9*).

**Table:** Estimated incubation periods for SARS-CoV-2 Omicron BA.5 variant according to population percentiles using lognormal, gamma, or Weibull probability distributions in study of epidemiology of SARS-CoV-2 Omicron BA.5 infections, Macau, June–July 2022*

Percentile	Incubation, d (95% Cl)		
	Lognormal	Gamma	Weibull
Mean	3.27 (3.07–3.45)	3.00 (2.83–3.16)	3.26 (3.07–3.40)
2.5th	1.46 (1.17–1.74)	1.87 (1.74–2.00)	1.10 (0.93–1.25)
5th	1.64 (1.37–1.90)	2.02 (1.89–2.16)	1.38 (1.20–1.52)
50th	3.04 (2.87–3.18)	2.95 (2.79–3.12)	3.23 (3.03–3.38)
95th	5.67 (4.97–6.67)	4.13 (3.94–4.33)	5.24 (4.74–5.52)
97.5th	6.39 (5.49–7.77)	4.39 (4.19–4.59)	5.61 (5.05–5.94)
99th	7.34 (6.16–9.29)	4.70 (4.49–4.91)	6.03 (5.40–6.44)
AIC value†	360.80	377.57	370.45

*Mean number of days was estimated after adjusting for exponential growth. AIC, Akaike information criterion. †AIC values were calculated to compare the fit of each model.

Among 572 PCR-confirmed cases reported by June 30, a total of 23 case-patients had received 1 SARS-CoV-2 vaccine dose, 216 had received 2 vaccine doses, 224 had received 3 vaccine doses, and 109 were unvaccinated (Appendix Table 3). Although only 10% of the population was unvaccinated, 19% of the SARS-CoV-2–infected persons were unvaccinated.

Among the 1,821 locally infected case-patients, 6 deaths occurred (Appendix Table 4). Therefore, the case-fatality risk for Omicron-infected persons in Macau was 0.33% (95% CI 0.13%–0.76%). Persons who died of COVID-19 were 86–100 (mean 92.5, SD +5.0) years of age. Among the 6 case-patients who died, 3 had received 2 doses of vaccine, and the other 3 were unvaccinated.

A limitation of our study is that we could not separate the effects of each public health and social measure because they were implemented as a package. High compliance with those stringent measures during the outbreak might have maximized the effectiveness of the interventions (*10*), although those measures might not be easily applicable to other locations outside of China. In conclusion, our study indicates that SARS-CoV-2 outbreaks can be controlled through stringent public health and social measures, such as repeated universal PCR testing and stay-at-home orders lasting at least 2 weeks.

Appendix Additional information for epidemiology of SARS-CoV-2 Omicron BA.5 infections, Macau, June–July 2022.
