# Supplementary material for: Epidemiology of SARS-CoV-2 Omicron BA.5 Infections, Macau, June–July 2022
Source: Emerg Infect Dis. 2023 Feb;29(2):453–6. doi: 10.3201/eid2902.221243 (PMC9881764; doi:10.3201/eid2902.221243)
Supplement: Appendix — Additional information for epidemiology of SARS-CoV-2 Omicron BA.5 infections, Macau, June–July 2022. [file 22-1243-Techapp-s1.pdf]

Article DOI: https://doi.org/10.3201/eid2902.221243

Epidemiology of SARS-CoV-2 Omicron BA.5 Infections, Macau, June–July 2022

Appendix

Materials and Methods

Incubation Period Estimation

We obtained person-level data for the first 500 COVID-19 cases diagnosed in Macau during June 18–29, 2022 (https://www.ssm.gov.mo/apps1/PreventCOVID-19/en.aspx#clg22916). We used symptom keywords (feel unwell, fever, fatigue, sore throat, cough, stuffy nose, runny nose, diarrhea, headache, chills) to filter cases for onset date information. For each case, we assumed the following behaviors as possible exposure periods: having contact with persons with confirmed COVID-19, visiting red or yellow code zone(s), and dining outside of the home. If none of those high-risk behaviors were mentioned in the case report, we assumed that the person was infected during other lower-risk outdoor activities, such as shopping or walking, and the periods of lower-risk outdoor activities were treated as potential exposure periods. For each case, we recorded the start and end time of the exposure period. For cases with >1 possible exposure period, we processed each possible exposure time identically. Those data were considered as interval-censored data in the estimation.

We fitted lognormal, gamma, and Weibull parametric probability distribution models to the data to estimate incubation periods by using the maximum likelihood method (*1*). The best fitted model was determined by the smallest value of the Akaike information criterion. We derived estimates of means, SD, medians, and 95% percentiles for incubation period distribution from the models. We determined the corresponding 95% CIs for each estimate by using the parametric bootstrap method with 1,000 bootstrapped samples. In our calculations, we corrected sampling bias according to the computed exponential growth rate (r = 0.18) of the Omicron BA.5 variant by using data collected from the first 500 COVID-19 cases during June 18–29, 2022.

Estimate of Time-Varying Reproductive Number

Model details

We estimated the time-varying reproductive number (R_t) from real data as previously described (*2*). In brief, the R_t assumes that the distribution of infectiousness through time is independent of calendar time. Transmission is then modeled by using a Poisson process. The probability distribution $w(s)$ denotes the infectiousness profile since infection; therefore, the rate for infection at time step t–s generates new infections in time step t that are equal to $R(t)w(s)$, where $R(t)$ is the instantaneous reproductive number at time t. The actual number of new local infection cases at time t was denoted as $Y(t)$; the infection incidence at time t has Poisson distribution with mean $R(t)\sum_{s=1}^{t} Y(t-s)w(s)$.

The true number of infection cases was unobserved because we only observed new local cases reported on day k [$Y(k)$]. To overcome this challenge, we used:

$$Y(t) \sim \text{Poisson}\{R(t)\sum_{k=1}^{t-1} Y(k)w(t-k)\}$$

where $R(t)$ is the time-varying effective reproductive number at time t.

Likelihood function

We used the smoothing method as previously described (*2*), assuming that the transmissibility was constant over a time period $[t-\tau+1, t]$, where τ is the smoothing parameter. Hence, likelihood of infection incidence during this time period was:

$$P\big(Y(t), \ldots, Y(t-\tau+1)\big|Y(1), \ldots Y(t-\tau)\big) = \prod_{s=t-\tau+1}^{t} \frac{\big(R^{\tau}(t)\phi(s)\big)^{Y(s)} e^{-R^{\tau}(t)\phi(s)}}{Y(s)!}$$

where $\phi(s) = \sum_{k=1}^{s-1} Y(k)w(s-k)$. The total likelihood is the product of the individual likelihood at each time t in the observed data. The first $\tau - 1$ days were excluded because of τ-day smoothing.

Prior Probability Distribution

We assumed the prior probability distribution for R(t) was a gamma distribution (parameters 1, 5) (mean 5, SD $\pm$5).

Estimate of Model Parameters

We conducted our analysis in a Bayesian framework and used the Markov chain Monte Carlo method (*3*) to estimate model parameters. At each step k, we updated the model parameters θ by using the random walk Metropolis-Hastings algorithm (*3*). The step size of the proposal was adjusted to have an acceptance rate of 20%–30%.

Assumptions for Input Parameters

For incubation period distribution, we use the estimated distribution mean of 3.2 days. The empirical distribution of delay from disease onset to report was set as follows: 50% probability delayed by day 1, 40% by day 2, and 8% by day 3, then the remaining 2% probability was equally distributed over days 4–20. According to those distributions, we constructed the distribution for delay from initial infection to report by convoluting the incubation period distribution and empirical distribution of delay from onset to report. We then used a deconvolution approach (*4*) to obtain the epidemic curve by infection time, which was achieved by using the fit incidence function in the incidental package in R (The R Project for Statistical Computing, https://www.r-project.org).

The infectiousness since infection w_t was a convolution of incubation period and infectiousness relative to onset (allowed to be presymptomatic) according to viral shedding data with a shifted gamma distribution (mean 12.3 days, SD $\pm$8 days) (*5*); therefore, the shift parameter c was 12.3 days. Peak infectiousness was on day 0. We proposed an infectiousness profile $g_c(t_l - t_{sl})$ that described the probability of a transmission event occurring at time t_l after illness onset t_{sl}. We assumed a gamma distribution g(t) with a time shift to determine the start of infectiousness c days before symptom onset; therefore, $g_c(t) = g(t + c)$ determined presymptomatic transmission. We analyzed the epidemic curve until July 31, 2022, and used $\tau = 7$ in our analysis to avoid unstable estimates for time-varying reproductive numbers.

Inference

After obtaining the epidemic curve by infection time, we used the Markov chain Monte Carlo approach (*3*) to estimate R_t. We accounted for the uncertainty of input parameters, including incubation period and infectiousness profile, to obtain the final estimates of R_t as follows. We used the bootstrap approach (*6*,*7*) to account for input parameter uncertainty and obtain final estimates of R_t. In each iteration, we used the deconvolution approach described

previously to reconstruct the epidemic curve by infection dates and estimate R_t. We determined the mean and 2.5% and 97.5% quantiles for those R_t estimates for each time point across the 200 bootstrap iterations.

References

1. Verity R, Okell LC, Dorigatti I, Winskill P, Whittaker C, Imai N, et al. Estimates of the severity of coronavirus disease 2019: a model-based analysis. Lancet Infect Dis. 2020;20:669–77. PubMed https://doi.org/10.1016/S1473-3099(20)30243-7

2. Cori A, Ferguson NM, Fraser C, Cauchemez S. A new framework and software to estimate time-varying reproduction numbers during epidemics. Am J Epidemiol. 2013;178:1505–12. PubMed https://doi.org/10.1093/aje/kwt133

3. Bennet JE, Berzuini C, Best NG, Buck C, Carlin BP, Clayton DG, et al. Markov chain Monte Carlo in Practice. In: Gilks WR, Richardson S, Spiegelhalter DJ, editors. London: Chapman & Hall; 1996. p. 1–512.

4. Miller AC, Hannah LA, Futoma J, Foti NJ, Fox EB, D'Amour A, et al. Statistical deconvolution for inference of infection time series. Epidemiology. 2022;33:470–9. PubMed https://doi.org/10.1097/EDE.0000000000001495

5. He X, Lau EHY, Wu P, Deng X, Wang J, Hao X, et al. Temporal dynamics in viral shedding and transmissibility of COVID-19. Nat Med. 2020;26:672–5. PubMed https://doi.org/10.1038/s41591-020-0869-5

6. Salje H, Cummings DAT, Rodriguez-Barraquer I, Katzelnick LC, Lessler J, Klungthong C, et al. Reconstruction of antibody dynamics and infection histories to evaluate dengue risk. Nature. 2018;557:719–23. PubMed https://doi.org/10.1038/s41586-018-0157-4

7. Tsang TK, Wu P, Lau EHY, Cowling BJ. Accounting for imported cases in estimating the time-varying reproductive number of coronavirus disease 2019 in Hong Kong. J Infect Dis. 2021;224:783–7. PubMed https://doi.org/10.1093/infdis/jiab299

Appendix Table 1. Public health and social measures implemented during June–July 2022 in Macau in study of epidemiology of SARS-CoV-2 Omicron BA.5 infections*

Date	Type	Subtype	Measure	Date announced	Source
2022 Jun 15	Travel	Border control	Period of centralized medical observation in isolation for arrivals shortened to 10 d beginning at 0:00 AM on June 15	2022 Jun 10	https://www.gcs.gov.mo/detail/en/N22FK5iS6F?14
2022 Jun 19	Community	Quarantine	Zone-specific, multi-level focused approach to epidemic prevention and control activated. Closure and control measures implemented for living areas of persons with confirmed cases. Nucleic acid tests will temporarily be conducted on days 1–3, 5, and 7. Only entry into but no exit from the zone will be permitted with the exception of staff for red-coded zones. No person can leave the zone until the first test is concluded for yellow-coded zones.	2022 Jun 19	https://www.gcs.gov.mo/detail/en/N22FSgsvrf?6
2022 Jun 19	Clinical	Citywide NAT	Launch of first round of citywide NATs from 12:00 AM on June 19 to 12:00 PM on June 21	2022 Jun 19	https://www.gcs.gov.mo/detail/en/N22FSuIQCY?8
2022 Jun 21	Community	Health code application	Enabled the risk check function of Macau Health Code	2022 Jun 21	https://www.gcs.gov.mo/detail/en/N22FUNLza4?30
2022 Jun 22	Clinical	RAT	All residents in Macau were required to conduct RATs.	2022 Jun 22	https://www.gcs.gov.mo/detail/en/N22FUAMVEq?39
2022 Jun 22	Clinical	Focused NAT	Launch of NATs in key areas for key groups. Inspection focus of key groups included persons who work or live in Macao with Myanmar passports and those who had the same action tracking as positive cases during the last citywide NAT program. Inspection focus in key areas on June 22 included persons who live, work, or have activities in area surrounded by Avenida de Horta e Costa, Rua do Almirante Costa Cabral, Estrada Do Repouso, and Avenida do Almirante Lacerda. The focus of NATs for key areas on June 25 included persons who worked or stayed at the following place(s) for >30 min after June 18: Luís de Camões Park, Lou Lim Ioc Park, shops at Rua da Emenda, shops along Bairro Iao Hon 1st Street to Bairro Iao Hon 8th Street, Fu Tai Industrial Building on Avenida de Venceslau de Morais, and San Kin Yip Commercial Centre on Avenida da Amizade.	2022 Jun 22	https://www.gcs.gov.mo/detail/en/N22FU62sfT?4
2022 Jun 23	Clinical	Citywide NAT	Launch of second round of citywide NATs from 9:00 AM on June 23 to 12:00 PM on June 24	2022 Jun 22	https://www.gcs.gov.mo/detail/en-hans/N22FVAAHVa?17
2022 Jun 23	Community	Restaurant	Certain recreational facilities and dining-in at restaurants suspended beginning at 5 PM on June 23	2022 Jun 23	https://www.gcs.gov.mo/detail/en/N22FWwg05w?9
2022 Jun 25	Clinical	Focused NAT	Launch of NAT for key areas. Focus of NATs for key areas included persons who worked or stayed at the following place(s) for >30 min after June 18: Luís de Camões Park, Lou Lim Ioc Park, shops at Rua da Emenda and Bairro Iao Hon 1st Street to Bairro Iao Hon 8th Street, Fu Tai Industrial Building on Avenida de Venceslau de Morais, and San Kin Yip Commercial Centre on Avenida da Amizade.	2022 Jun 25	https://www.gcs.gov.mo/detail/en/N22FYNcgqW?keyword = key+areas+nucleic
2022 Jun 25	Community	Elderly residential care home	Implemented a preventive closed-loop management in elderly residential care homes	2022 Jun 24	https://www.gcs.gov.mo/detail/zh-hans/N22FX5iTTD?6
2022 Jun 27	Clinical	RAT	Beginning on June 27, citizens must perform a self-rapid antigen test and declare before going to the inspection station. If the test result was negative, they could go to the inspection station for a NAT and show the certificate; otherwise, they could not enter the station.	2022 Jun 26	https://www.gcs.gov.mo/detail/zh-hans/N22FZ0BVyB?3
2022 Jun 27	Clinical	Citywide NAT	Launch of citywide NATs from 9:00 AM on June 27 to 12:00 PM on 28 June 28. The 3rd round of NAT was June 27–28.	2022 Jun 26	https://www.gcs.gov.mo/detail/en/N22FSuIQCY?8
2022 Jun 29	Clinical	RAT	Announced that RAT results affect the health code and launched requirement for RATs for 2 consecutive days starting on June 29–30. If citizens did not complete the declaration of RAT results as required, their Macau health code would be converted to a yellow code at 0:00 AM the next day, and citizens must complete the test on the	2022 Jun 28	https://www.gcs.gov.mo/detail/en/N22FblvTVG?5

Date	Type	Subtype	Measure	Date announced	Source
			same day before they could be converted back to a green code. If the RAT was not done for 2 consecutive days, the code would be changed to red, and NAT would be required. The code could be changed back to green after a negative NAT.		
2022 Jul 4	Clinical	Citywide NAT	Launch of citywide NAT from 9:00 AM July 4 to 6:00 PM on July 9. The 4th round of NAT was July 4-5, 5th round was July 6-7, and 6th round was July 8-9.	2022 Jul 3	https://www.gcs.gov.mo/detail/en/N22GCad4aM?9
2022 Jul 9	Community	Relatively static management	Macao suspended all non-essential industrial and commercial activities from 0:00 AM on July 11 to 0:00 AM on July 18 and called on residents to minimize movement to reduce risk of virus transmission, except for those persons whose activity was deemed essential to the community and day-to-day lives of the public.	2022 Jul 9	https://www.gcs.gov.mo/detail/zh-hans/N22GIkjA4P?10
2022 Jul 10	Clinical	Citywide NAT	Launched 4 more rounds of citywide NATs from 9:00 AM on July 10 to 6:00 PM on July 17. The 7th round of NAT was July 10–11, 8th round was July 12–13, 9th round was July 14–15, and 10th round was July 16–17.	2022 Jul 8	https://www.gcs.gov.mo/detail/zh-hans/N22GHvwnRa?7
2022 Jul 18	Clinical	Citywide NAT	Launched 3 more rounds of citywide NATs from 9:00 AM on July 18 to 6:00 PM on July 23. The11th round of NAT was July 18–19, 12th round was July 20–21, and 13th round was July 22–23.	2022 Jul 16	https://www.gcs.gov.mo/detail/zh-hans/N22GPkhUgx?8
2022 Jul 30	Clinical	Citywide NAT	Launched 14th round of citywide NAT from 9:00 AM on July 30 to 6:00 PM on 31 July 31	2022 Jul 20	https://www.gcs.gov.mo/detail/zh-hans/N22GTdO030?11

*NAT, nucleic acid test; RAT, rapid antigen test.

Appendix Table 2. Number of persons tested during multiple rounds of citywide nucleic acid testing in study of epidemiology of SARS-CoV-2 Omicron BA.5 infections, Macau, June–July 2022*

Round	Dates	No. tested	Proportion, %
1	Jun 19–21	677,586	99.21
2	Jun 23–24	667,144	97.68
3	Jun 27–28	652,544	95.54
4	Jul 4–5	637,349	93.32
5	Jul 6–7	667,597	97.74
6	Jul 8–9	658,906	96.47
7	Jul 10–11	658,879	96.47
8	Jul 12–13	665,385	97.42
9	Jul 14–15	664,532	97.30
10	Jul 16–17	667,342	97.71
11	Jul 18–19	666,849	97.64
12	Jul 20–21	669,234	97.98
13	Jul 22–23	722,335	105.76
14	Jul 30–31	707,277	103.55

*Dates for 14 rounds of testing by PCR and numbers and percentages of persons tested during each round.

Appendix Table 3. Vaccination status among general population and persons with confirmed COVID-19 cases in June 2022 in study of epidemiology of SARS-CoV-2 Omicron BA.5 infections, Macau, June–July 2022

	General population on 2022 Jun 19	Confirmed cases during 2022 Jun 19–30
Type	No. persons (%)	No. persons (%)
Unvaccinated	70,923 (10.38)	109 (19.06)
Received only the 1st dose	27,701 (4.05)	23 (4.02)
Completed the 2nd dose	307,851 (45.06)	216 (37.76)
Completed the 3rd dose	276,725 (40.50)	224 (39.16)
Received at least 1 dose	612,277 (89.62)	463 (80.94)
Received at least 2 doses	584,576 (85.56)	440 (76.92)
Total	683,200	572

Appendix Table 4. Characteristics of the 6 patients who died of COVID-19 in Macau in study of epidemiology of SARS-CoV-2 Omicron BA.5 infections, Macau, June–July 2022

Date of hospital admission	Date of death	Age, y/sex	Vaccination status	Chronic disease history
2022 Jun 29	2022 Jul 3	94/F	2 doses of inactivated virus vaccine	Hypertension, hyperlipidemia,stroke
2022 Jun 30	2022 Jul 3	100/F	None	Hypertension,brain degeneration, fractures
2022 Jul 6*	2022 Jul 12	88/F	Unknown	Severe diabetes, heart disease, aortic dissection tear
2022 Jul 3*	2022 Jul 13	94/F	Unknown	Chronic heart failure, respiratory failure
2022 Jul 5	2022 Jul 14	86/F	2 doses of inactivated vaccine	Chronic kidney disease, dementia
2022 Jul 9	2022 Jul 21	93/M	None	Chronic heart disease, chronic lung disease

*Macau Health Bureau did not report detailed vaccination status for these 2 patients. One patient received 2 vaccine doses, the other was unvaccinated.

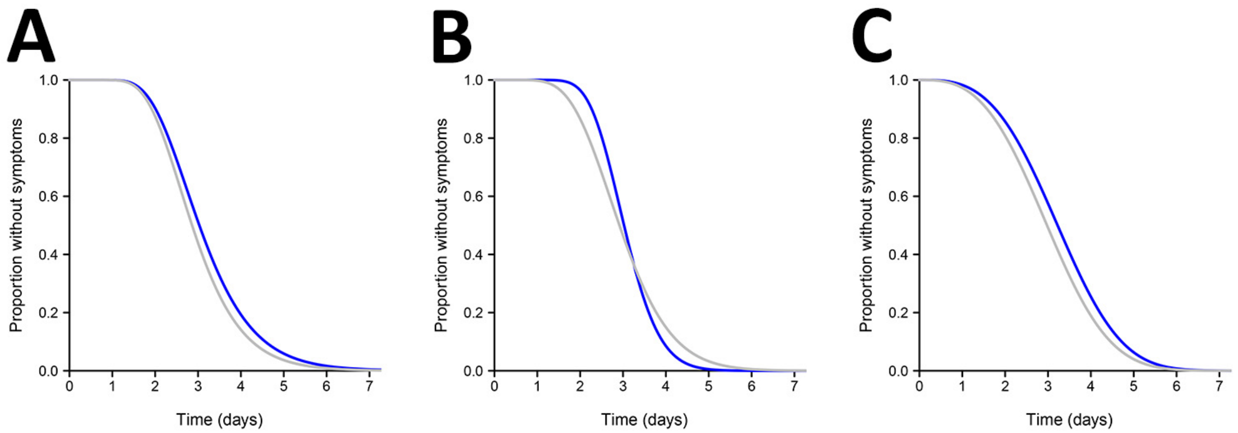

Appendix Figure. Estimated incubation period distributions for SARS-CoV-2 infections in study of epidemiology of SARS-CoV-2 Omicron BA.5 infections, Macau, June–July 2022. Gray lines show the cumulative probability for each distribution; blue lines show the cumulative probability after adjusting for integrations for each distribution. Data were fitted to (A) lognormal, (B) gamma, and (C) Weibull parametric probability distribution models to estimate incubation periods by using the maximum likelihood method.
